# Follicular development during the follicular phase of the menstrual cycle: the enigma of luteinizing hormone receptor function

**DOI:** 10.3389/fendo.2026.1817405

**Published:** 2026-06-09

**Authors:** Claus Yding Andersen, Liv la Cour Poulsen, Marie Louise Wissing, Malene Louise Johannsen

**Affiliations:** 1 The Fertility Clinic, Copenhagen University Hospital Herlev, Herlev, Denmark; 2 Institute of Clinical Medicine, Faculty of Health and Medical Sciences, University of Copenhagen, Copenhagen, Denmark; 3 Department of Urology, Copenhagen University Hospital - Herlev and Gentofte Hospital, Herlev, Denmark; 4 Alleris Fertility, Søborg, Denmark; 5 Drug Toxicology, Metabolism and Analysis Group, Department of Pharmacy, Faculty of Health and Medical Sciences, University of Copenhagen, Copenhagen, Denmark

**Keywords:** folliculogenesis, granulosa cells (GCs), human chorionic gonadotropin (hCG), luteinizing hormone receptor (LHR), ovarian stimulation (OS), theca cells (TCs)

## Abstract

**Background:**

The luteinizing hormone receptor (LHR) plays a pivotal role in regulating human follicular development and steroidogenesis through temporal and cell-specific expression on theca (TCs) and granulosa cells (GCs). Although LH-like activity (i.e., LH and/or human chorionic gonadotropin (hCG)) has long been incorporated into ovarian stimulation (OS) regimens, its precise physiological role in human folliculogenesis remains poorly defined. Recent large randomised clinical trials (RCT) have failed to demonstrate a consistent benefit of exogenous LH-like activity during OS, suggesting incomplete understanding of LHR-mediated actions in normo-gonadotropic women.

**Objective:**

To provide a new mechanistic interpretation of the published data from the Rainbow RCT (Fernández Sánchez et al., 2022 (9)), which evaluated the effect of increasing doses of recombinant hCG (rhCG) as LH-like activity co-administration during OS, and to formulate a hypothesis that explains its divergent hormonal responses and reproductive outcomes.

**Outcome of the Rainbow trial:**

In the Rainbow RCT, increasing doses of (rhCG) added to recombinant FSH (rFSH, i.e., rFSH-Δ) resulted in a dose-dependent reduction in the number of good-quality blastocysts and ongoing pregnancy rates, as well as paradoxical endocrine patterns. Androstenedione, 17-OH-progesterone (17OH-P_4_), testosterone, and oestradiol (E_2_) increased dose-dependently, whereas progesterone (P_4_), inhibin-A, and inhibin-B declined.

**Hypothesis:**

These findings suggest attenuation of GC function exerted by rhCG despite preserved or enhanced TC activity. The divergent hormonal responses observed reflect fundamental differences in LHR expression, density, and downstream signalling between GCs and TCs. During OS, FSH induces LHR expression in GCs, potentially leading to distinct receptor clustering and susceptibility to biased agonism by different LH or hCG isoforms. Constant exposure to rhCG—as opposed to the physiological pulsatile LH pattern—may further alter receptor dynamics, contributing to selective attenuation of GC-function whilst maintaining TC responsiveness.

**Conclusion:**

The Rainbow RCT may offer the first *in vivo* evidence supporting a temporal and cell type–specific difference in LHR function in human TCs and GCs. These data call for renewed investigation into LHR regulation, glycosylation, and receptor density in human immature GC and TC under both natural and stimulated conditions. Clarifying these mechanisms will be essential to optimizing gonadotropin combinations for OS and to advancing understanding of ovarian physiology.

**Clinical Trial Registration:**

https://www.clinicaltrialsregister.eu/ctr-search/trial/2017-003810-13/results, identifier 2017-003810-13.

## Introduction

The functional units of the ovaries are follicles, each containing one oocyte and granulosa cells (GCs) enclosed by a basal membrane and encircled by theca cells (TCs). Over 4–6 months, primordial follicles progress through developmental stages to form preovulatory follicles capable of releasing fertilizable oocytes during ovulation (1). Folliculogenesis is orchestrated by complex endocrine and paracrine interactions, primarily regulated by follicle-stimulating hormone (FSH) and luteinizing hormone (LH), acting through their respective receptors (FSHR and LHR). These hormones drive follicular growth, steroidogenesis, and temporal and cell type–specific functions within the follicle.

Ovarian stimulation (OS) is a key component of *in vitro* fertilization (IVF) treatment, designed to increase the number of preovulatory follicles to retrieve multiple oocytes. Gonadotropin preparations containing both FSH and LH-like activity (i.e., LH or human chorionic gonadotropin, hCG) have been widely used for decades (e.g., Menopur^®^ (Ferring), Meriofert^®^ (IBSA), and Pergoveris^®^ (Merck)). However, a recent review summarizing 30 years of clinical data found no consistent evidence that LH-like activity improves IVF outcomes, despite its recognised physiological importance in folliculogenesis (2).

The limited understanding of LH function and LHR activation in human follicles, either *in vivo* or *in vitro*, arises from several factors:

1. Isolated immature human GCs rapidly change and luteinise in culture, which entails initial downregulating of LHR with subsequent upregulation of endogenous LHR expression whilst acquiring high progesterone (P_4_)-secreting capacity.
2. *In vivo*, LHR is exposed to pulsatile LH release at 60–90-min intervals, whereas constant exposure leads to receptor downregulation or altered signalling (3).
3. LHR expression differs temporally and spatially between cell types: it is constitutive expressed in TCs but develops in GCs only after follicular selection (i.e., 8–10 mm) in normal women. Theca cell LHR distribution may vary during follicle maturation (4, 5).
4. Theca cell androgen synthesis depends on GC-derived growth factors such as IGFs and inhibin’s induced by FSH and which acts in synergy with LH stimulating LHR activity (6–8).

Given these complexities in studying LHR function during the follicular phase of the menstrual cycle, well-designed RCTs in women provide information on the *in vivo* effects of administration of LH-like activity and LHR activation. The recent Rainbow RCT evaluated recombinant FSH (i.e., rFSH-Δ) combined with increasing doses of recombinant hCG (rhCG) as the LH-like component (9) and provided an opportunity to evaluate LH-like effects on immature human GCs *in vivo*.

Unexpectedly, increasing rhCG doses were associated with unfavourable endocrine profiles and poorer reproductive outcomes—including fewer good-quality blastocysts and lower pregnancy rates throughout the different dosing groups—compared with rFSH alone. Subsequent analyses of follicular fluid (FF) revealed dose-dependent alterations in steroid and growth factor concentrations (10, 11). Recent insights into the differential regulation of steroidogenesis in GCs and TCs provide information to explain these findings (12–14).

The aim of the present study was to reexamine the results of the Rainbow RCT and potentially to generate a hypothesis centred on differential LHR function between GCs and TCs. This hypothesis incorporates the concepts of biased agonism, receptor density, glycosylation differences, and stage-specific LHR expression, aiming to advance understanding of human follicular regulation and identify targets for future research.

## Results of the Rainbow trial

The Rainbow RCT tested a newly developed rhCG in combination with an rFSH (r-FSHΔ) product as control with the aim of mimicking a classical urine-derived human menopausal gonadotropin (hMG) product containing both FSH and LH-like activity (9). It was conducted as a placebo-controlled, double-blind RCT in which women underwent OS in the long GnRH agonist protocol (9). Participants were randomised to receive either placebo or 1, 2, 4, 8, or 12 µg rhCG added (1 µg corresponds to approximately 30–31 IU) (10) to the daily individualised rFSH dose during OS. Number of good-quality blastocysts served as the primary endpoint. Thus, this trial qualifies by using the same FSH preparation in all dosing arms with only variations in the rhCG exposure.

Overall, 619 women (30–42 years) with anti-Müllerian hormone (AMH) levels between 5 and 35 pmol/L were randomised in equal proportions to the six treatment groups (≈100 women per arm). Final maturation of oocytes was performed by the administration of 250 µg rhCG (Ovitrelle) when 3 follicles ≥17 mm, but no more than 25 follicles ≥12 mm were reached.

**Figure 1:**
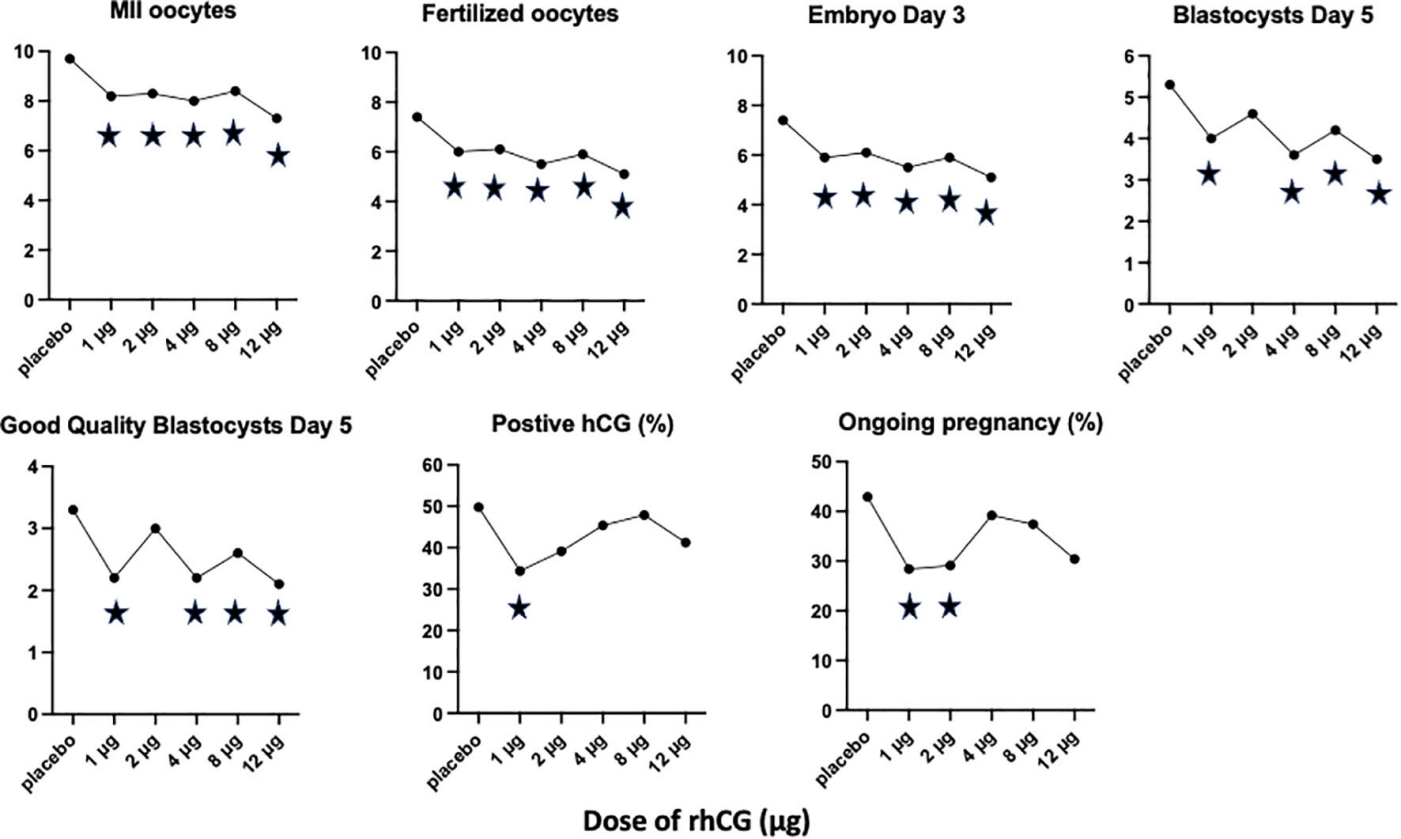
Outcome of ovarian stimulation and IVF treatment in women receiving different doses of rhCG (mean) in relation to the control group minus rhCG during the Rainbow RCT. Stars represent significant increases (p<0.05) between placebo and the group receiving rhCG. Data are from [9-11].

The results showed that the number of good-quality blastocysts was significantly reduced in all rhCG dose groups (except for one) as compared with placebo with rFSH only (**Figure 1**). The number of oocytes aspirated, and the ongoing pregnancy rate was numerically lower across all rhCG groups in relation to the placebo group with significantly lower pregnancy rates in two out of five groups (**Figure 1**). The number of follicles ≥17 mm was similar across groups (P < 0.10), whereas the group of follicles between 12 and 17 mm was reduced and significantly smaller in two of the five rhCG dosing groups (9).

**Figure 2:**
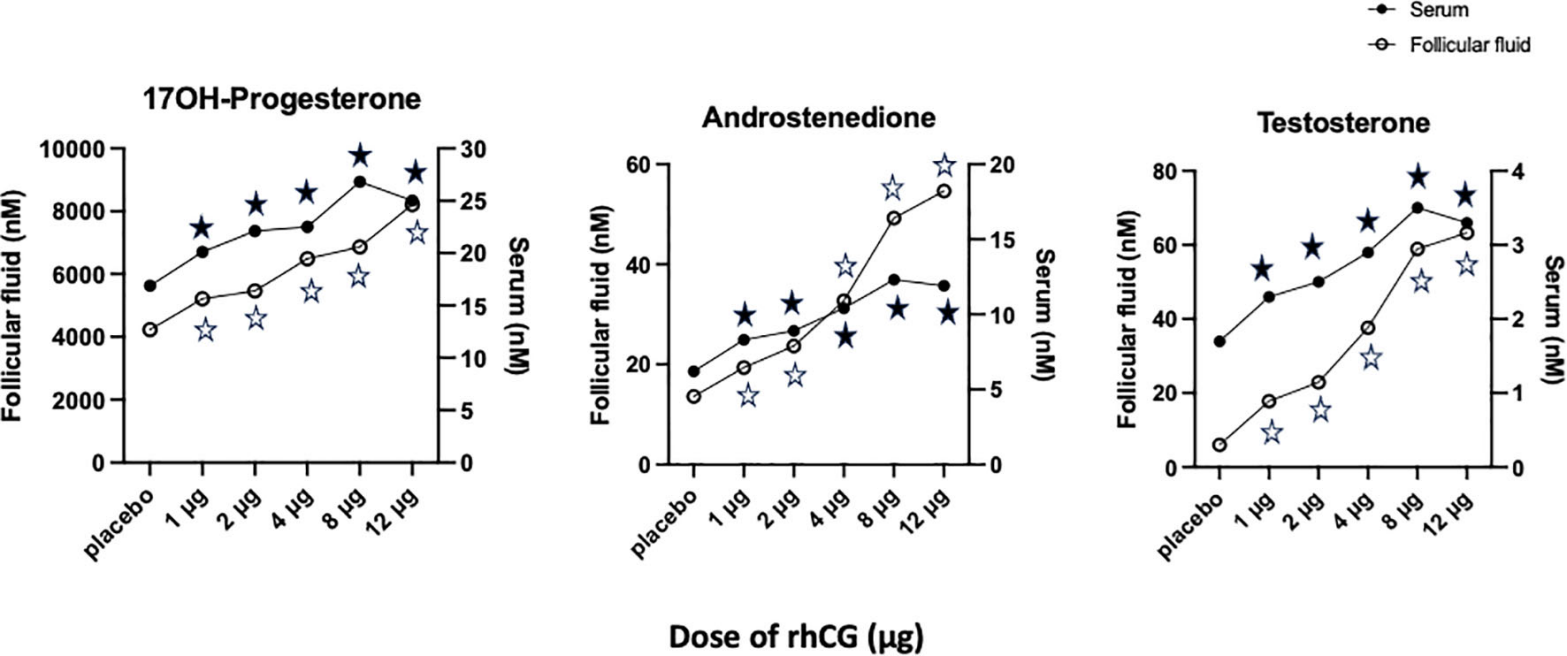
Concentrations of TC producing 17OH-P_4_, androstenedione, and testosterone in FF and in serum on the day of OPU in relation to dose of rhCG administered. Stars represent significant increases (p<0.05) between placebo and the group receiving different doses of rhCG in FF and circulation, respectively. Data are obtained from the Rainbow [9-11].

**Figure 3:**
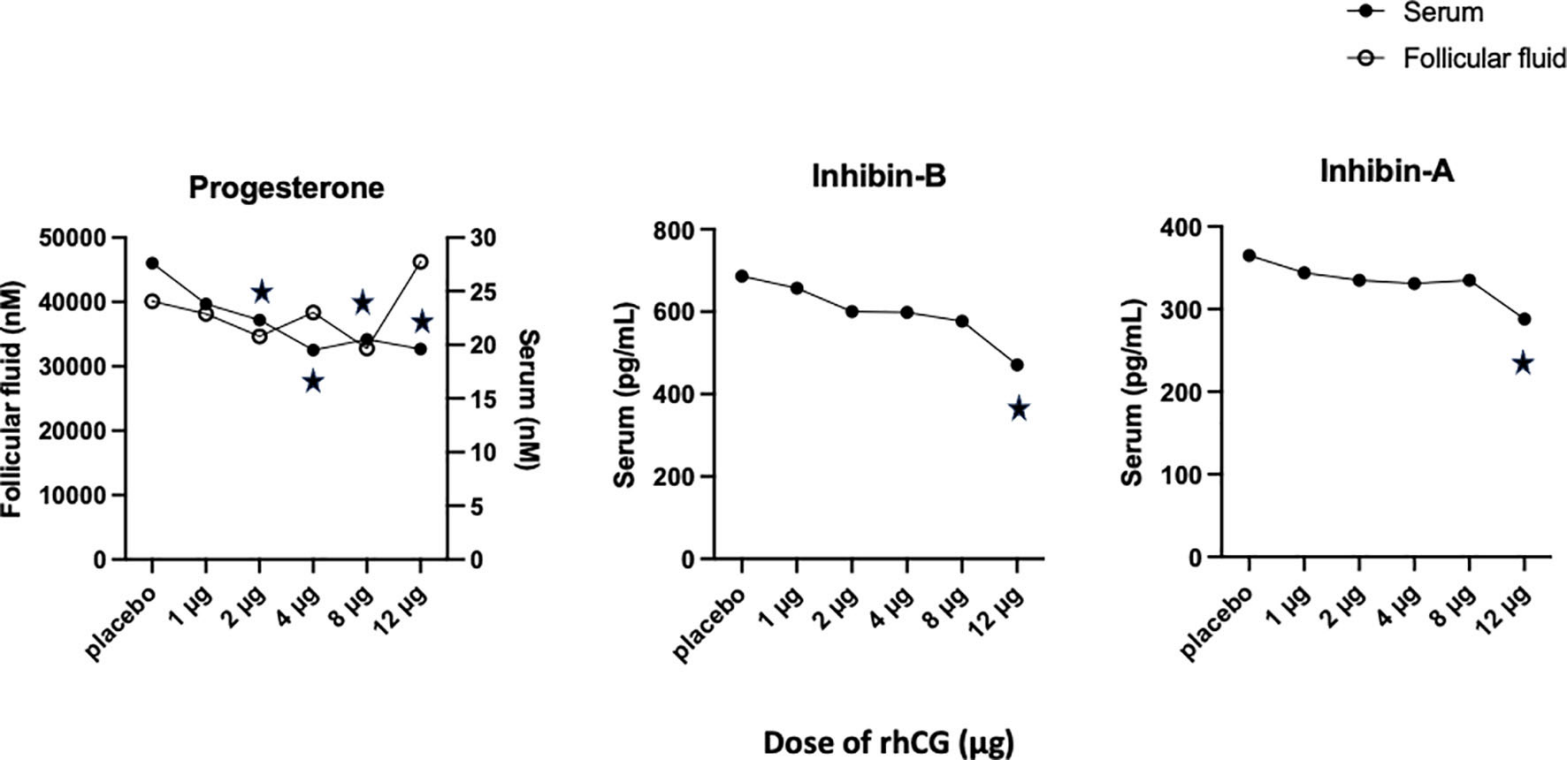
Concentrations of GC producing P_4_, inhibin-B, and inhibin-A in FF and in serum on the day of OPU in relation to dose of rhCG administered. Stars represent significant *reductions* (p<0.05) between placebo and the group receiving rhCG in FF and circulation. Data are obtained from the Rainbow RCT [9-11].

**Figure 4:**
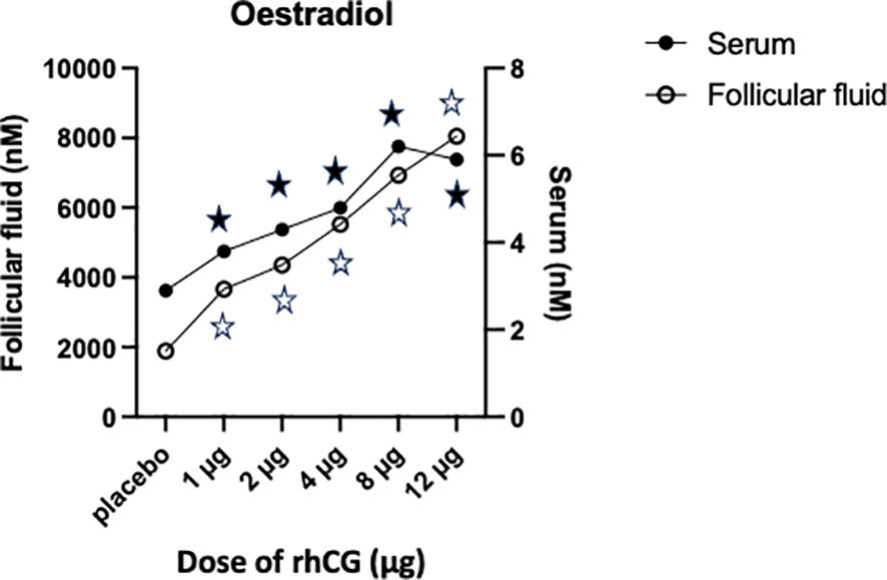
Concentration of GC producing E_2_ in FF and in serum on the day of OPU in relation to dose of rhCG administered. Stars represent significant increases (p<0.05) between placebo and the group receiving rhCG in FF and circulation. Induction of the enzyme converting testosterone into E_2_ is mainly induced by FSH acting directly on the GCs, and the increased production likely reflects increased substrate availability, which is suggested to be able to overcome a negative effect from the rhCG. Data are from [9-11].

Concentrations of oestradiol (E_2_), P_4_, 17-hydroxyprogesterone P_4_ (17OH-P_4_), androstenedione, testosterone, and inhibin-A in circulation and in FF were measured throughout the period of OS, whereas inhibin-B was only measured in serum at end-of-stimulation (**Figures 2**–**4**) (9–11).

## Human ovarian steroidogenesis

**Figure 5:**
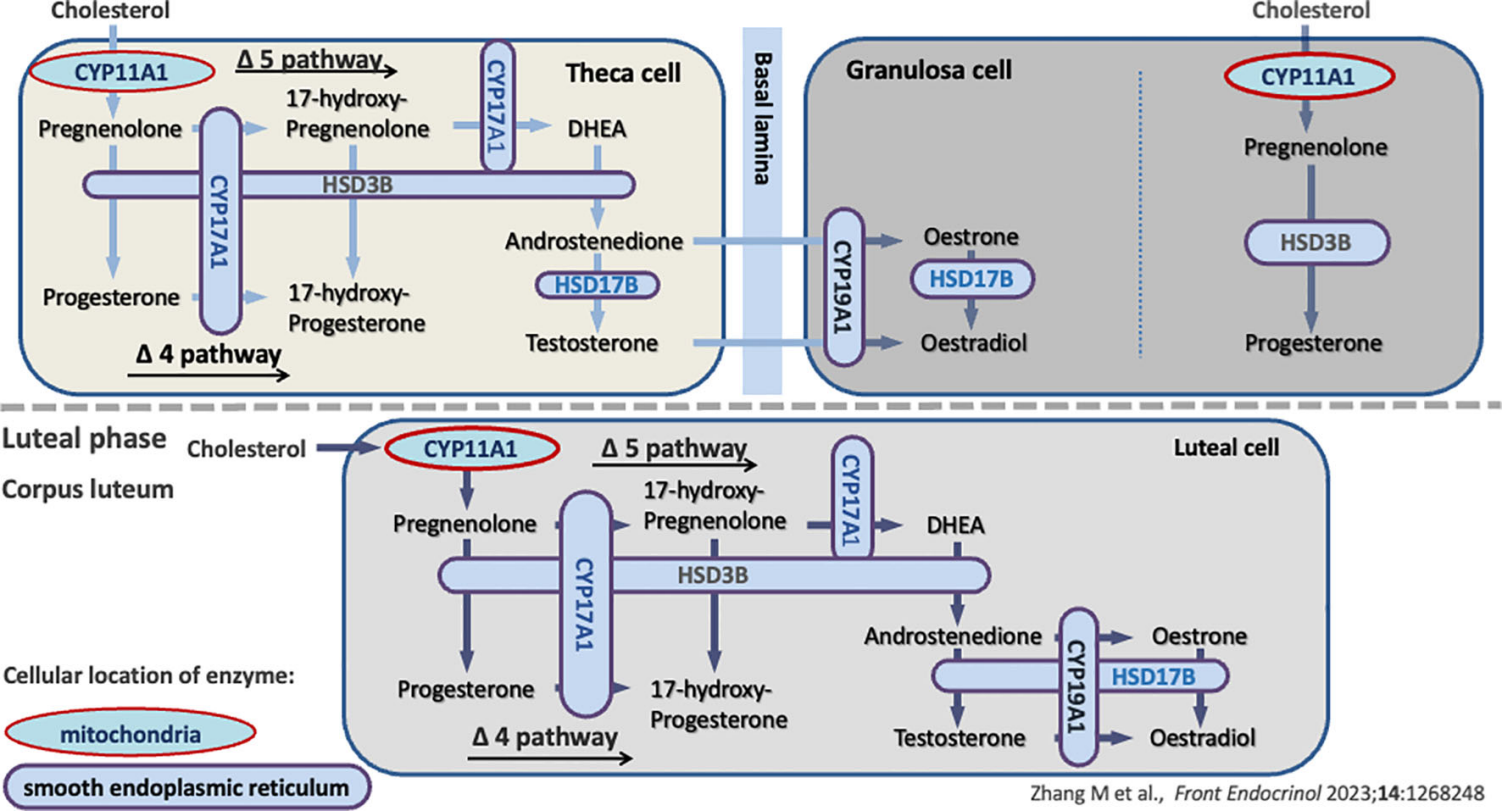
Human ovarian steroidogenesis. During the follicular phase, the TCs produce P_4_ and androgens, whereas GCs produce oestrogens and P_4_. During the luteal phase, luteal cells secrete androgens, P_4_, and oestrogens.Theca cells produce pregnenolone from cholesterol. The steroid biosynthesis then proceeds via either the Δ4 or Δ5 pathway. The Δ4 pathway converts pregnenolone to P_4_ and then 17OH-P_4_. In humans, the conversion of 17OH-P_4_ to androstenedione is limited. Through the Δ5 pathway, pregnenolone is metabolized into androstenedione or continues to be metabolized into testosterone before being delivered to GCs for aromatization into oestrogens (oestrone or E_2_). After ovulation, the GCs and TCs form the corpus luteum, which secretes all three hormones. [12]Abbreviations: CYP11A1, cytochrome P450 family 11 subfamily A member 1; CYP17A1, cytochrome P450 family 17 subfamily A member 1 (17α-hydroxylase/17, 20-lyase); CYP19A1, cytochrome P450 family 19 subfamily A member 1; HSD3B2, 3β-hydroxysteroid dehydrogenase 2; HSD17B, hydroxysteroid 17β dehydrogenase.

Human ovarian steroidogenesis is a complex process that involves an intricate interplay of various enzymes and metabolic pathways, with 3β-hydroxysteroid dehydrogenase 2 (HSD3B2) and cytochrome P450 family 17 subfamily A member 1 (17α-hydroxylase/17, 20-lyase (CYP17A1)) playing pivotal and cell-specific roles in the synthesis of P_4_ and 17-17OH-P_4_)(**Figure 5**) (12, 15, 16).

Importantly in understanding the Rainbow trial, recent studies have highlighted that ovarian steroidogenesis results in three primary terminal end products: 1) 17OH-P_4_ is produced by TCs throughout the follicular phase, 2) P_4_ is produced by GCs primarily after follicle selection and only secreted from TCs in limited amounts, and 3) E_2_ is converted in GCs from high concentrations of androgens produced in TCs—a process that also becomes upregulated upon follicle selection (**Figure 5**).

**Figure 6:**
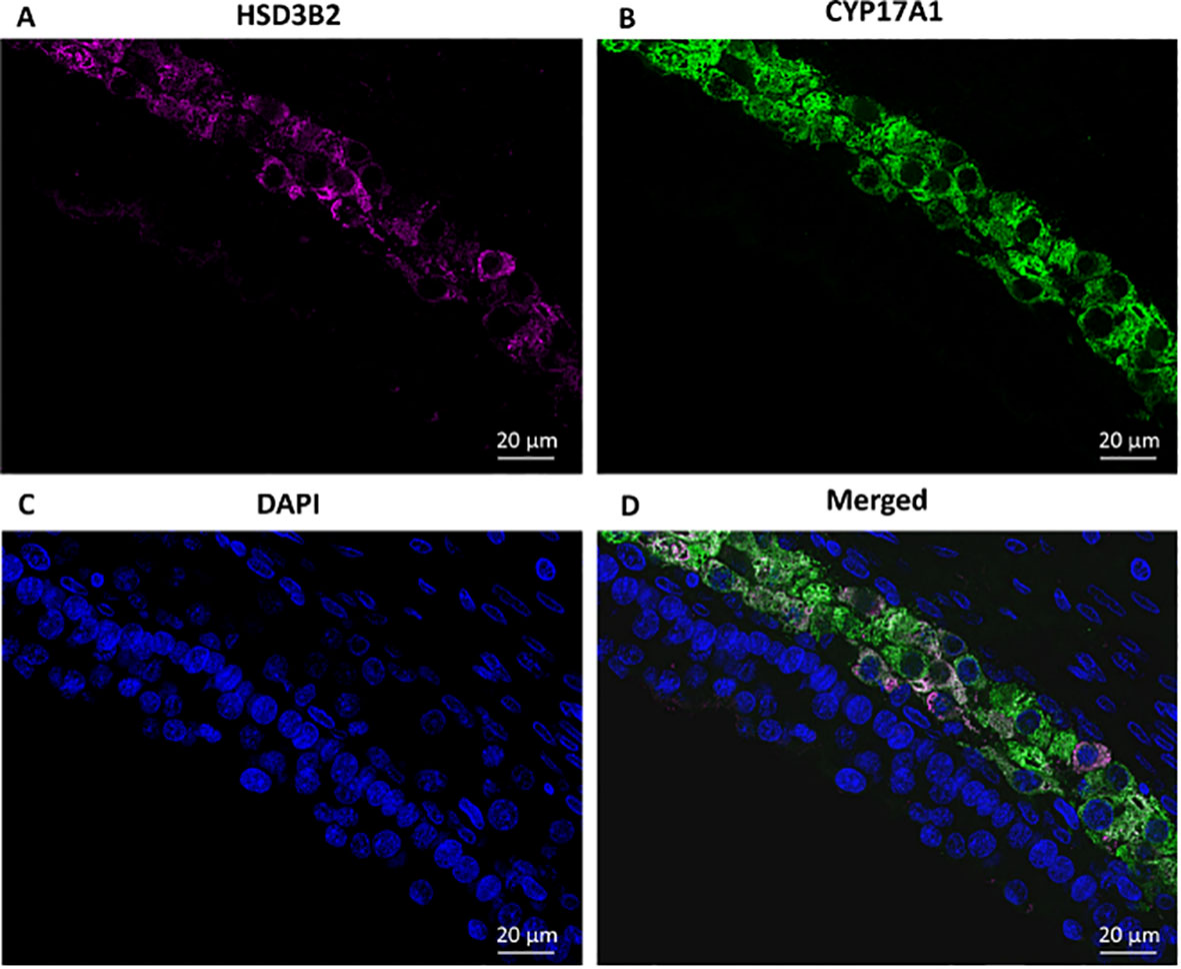
Immunofluorescence staining of human small antral follicles (5 mm diameter). **(A)** HSD3B2 immunoreactivity (magenta). **(B)** CYP17A1 immunoreactivity (green). **(C)** DAPI nuclear staining (blue). **(D)** Merged image showing colocalization of HSD3B2 and CYP17A1 in a subset of theca cells. HSD3B2 and CYP17A1 were exclusively detected in a subset of theca cells and consistently colocalized within the same cells. Scale bar = 20 µm.

The HSD3B2 enzyme catalyses the conversion of Δ5 to Δ4 metabolites, such as pregnenolone, into P_4_ (**Figure 5**). This reaction is significant because HSD3B2 lacks the ability to back-convert Δ4 metabolites to Δ5 forms, e.g., conversion of P_4_ back to pregnenolone (13, 15). In TCs, HSD3B2 expression begins at the early antral stage of approximately 0.2 to 0.3 mm in diameter (13). Notably, HSD3B2 always appears to be co-expressed with CYP17A1 (**Figure 6**) 13). This co-localization has important implications for steroidogenesis, as CYP17A1 converts P_4_ into 17OH-P_4_, thereby limiting the accumulation of P_4_ within TCs (12). In humans, CYP17A1 has minimal capacity to further metabolise 17OH-P_4_ into androstenedione (i.e., the lyase activity), making 17OH-P_4_ a terminal TC product (15) (**Figure 5**). In contrast, CYP17A1 actively catalyses the conversion of pregnenolone to 17OH-pregnelonone (i.e., hydroxylase activity) and dehydroepiandrosterone (DHEA) via the Δ5 pathway, contributing to subsequent androgen and E_2_ synthesis (**Figure 5**).

Interestingly, measurements of FF from small antral follicles (with diameters ranging from 3 to 13 mm) revealed that the concentration of 17OH-P_4_ is at least 10 times higher than that of P_4_ at this stage (13). This implies that 17OH-P_4_ becomes accumulated in FF from TCs and suggests that GCs are relatively quiescent undertaking P_4_ synthesis whereas TCs actively convert P_4_ into 17OH-P_4_ (12). Furthermore, the ratio of P_4_ to 17OH-P_4_ in circulation remains consistently below one throughout the follicular phase of the normal menstrual cycle and during OS with exogenous gonadotropins (13). Collectively, these data demonstrate that CYP17A1 only to a very limited extend support the conversion of 17OH-P_4_ to androstenedione in women. HSD3B2 are not expressed in GC in small antral follicles but become increasingly expressed after follicular selection (≈8–10 mm in diameter). The resulting P_4_ accumulates in FF, and concentrations of P_4_ in preovulatory FF reach very high concentrations of 30.000 nmol/L (17–19) and still only appear in circulation in concentrations of single-digit numbers.

After follicle selection, CYP19A1 become upregulated in GCs, where they initiate aromatization of androgens into oestrogens, resulting in increasing concentration of especially E_2_ in both FF and circulation.

## Expression LH receptor-biased agonist

Biological activity of LH is mediated via G-protein-coupled receptor proteins. The mature human LHR (hLHR) consists of 699 amino acids with a molecular weight of approximately 85 kDa, approximately 10 kDa of which is attributed to glycosylation (20, 21). The LHR is activated by either LH or hCG, triggering a plethora of intracellular signalling pathways including increased adenylate cyclase activity (cAMP) and activation of phospholipase C and other signalling cascades (22).

### Glycosylation and biased agonist

**Figure 7:**
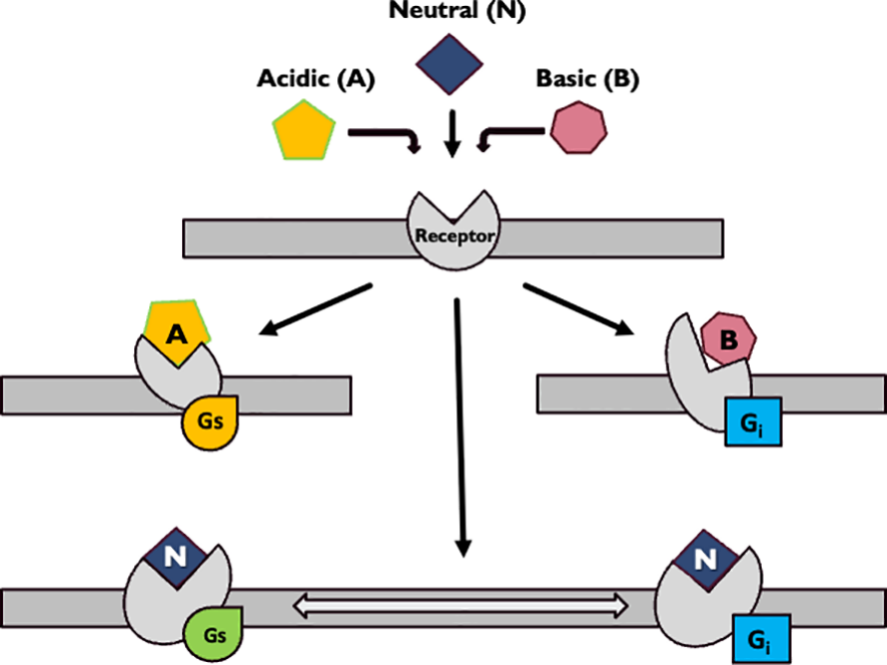
Isoforms of LH/hCG exist, as FSH molecules, with a range of isoelectric points and induce separate signalling properties that allow for an excessive number of signals to be transduced effectively via the LHR. This classify LH/hCG isoforms as biased agonists.

Both FSH and LH/hCG have considerable differences in their attached sugar residues. Pituitary release of different isoforms is mainly determined by the E_2_ concentration in circulation (23–25). Different isoforms with different glycosylation interact with receptors in different ways and induce different biological pathways (24, 26, 27). This phenomenon, known as biased agonism, explains how various LH/hCG isoforms induce distinct biological activities and signalling pathways (28–31)(**Figure 7**).

In connection with the Rainbow trial, the gonadotropins used (both rhCG and FSH-Δ) were expressed in human HEC-293 cells and showed different glycosylation profiles compared with recombinant gonadotropins expressed in the Chinese hamster ovary (CHO) cell line or compared with a urinary hCG (uhCG) (32, 33). Pharmacokinetic studies of the new rhCG showed in men an increased exposure and longer half-life resulting in higher testosterone concentrations as compared with rhCG developed in a CHO-cell line (33), suggesting differences in glycosylation patterns potentially leading to biased agonism (**Figure 7**).

Furthermore, the large N-terminus extracellular domain of the LHR with 340 amino acids is heavily glycosylated and contains six possible glycosylation sites (34, 35). Knowledge of the importance of LHR glycosylation for activity and signal transduction to target cells is, however, limited and based mainly on knockout animal studies performed mostly in rat and porcine (35). Human studies utilizing hLHR expression in human embryonic kidney cells (HEK-293) have shown that individual mutations of any one of the six motifs for glycosylation have only limited effect of hCG binding affinity or hCG-stimulated cAMP production (36). It has been speculated that the glycosylation has some importance for the receptor trafficking to the cell surface (34), and noticeably, it has been described that deletion of exon 10 in the LHR abolishes the activity of human LH but not hCG (37) and results in reduced density of cell surface receptors (38). However, overall, functional information on the hLHR remains limited and incomplete information on LHR characterization on human GCs and TCs in general and from the Rainbow trial is available.

### Receptor density and clustering

The density of LHR on the cell surface is a crucial determinant of its activity. Research in MA-10 cells (a clonal strain of mouse Leydig tumour cells) has demonstrated that increased receptor density (as measured by 125I-hCG binding) correlates positively with cAMP accumulation and P_4_ production in response to stimulation with hCG (22). Interestingly, these responses were not uniform; cAMP levels exhibited a biphasic dependence on receptor density, whereas P_4_ production showed a more linear relationship. This emphasises that receptor density is important in determining the activity of target cells and that receptor response depends on many parameters beyond substrate concentration and receptor density indicating the biased agonist nature of LH/hCG.

Additionally, receptor clustering and oligomerization, which may occur as dimers or larger complexes in the plasma membrane, appear to be essential for LHR activation (39–41). Upon ligand binding, clustering intensifies in a ligand concentration-dependent manner. Furthermore, activation of LHR may occur via cis-activation, where ligand-bound receptors directly initiate signalling, or trans-activation, where a ligand-bound receptor interacts with another receptor unable to bind the ligand to trigger signalling (41). The precise effects of the clustering and cis or trans activation are not clarified and have not been studied in immature human GCs, and no information from the Rainbow trial is available. However, it has been hypothesised to be involved in activation of signalling pathways, receptor desensitization, and internalization of LHRs following activation and represent other aspects of how LH or hCG stimulate target cells (41).

### Studies on human un-luteinised TCs and GCs

In GCs and TCs, LHR expression in human antral and preovulatory follicles has only been studied to a limited extent. Furthermore, LHR dynamics are likely to differ significantly between natural cycles and ovarian stimulation (OS). During OS, GCs are exposed to high levels of exogenous FSH, corresponding to that of the mid-cycle surge of gonadotropins averaging around 15–18 IU/L (42, 43). These high levels of FSH are likely to affect LHR expression on the GCs differently compared with natural cycles (44–46). Conversely, TCs often experience lower LH-like activity during OS than during natural cycles due to pituitary downregulation with GnRH analogues, which indeed was the case in the Rainbow trial which used long agonist downregulation protocol and exposed the TCs of only very low levels of endogenous LH activity (9).

### Studies on LHR function and activity

Noticeably, most studies on LHR have been performed with animal cells or cell lines, which has been genetically manipulated to express LHR. However, the LHR density is seldom reported. Many studies have been performed with the HEK-293 cell line where the LHR is cloned and expressed, but this cell line is chromosomally abnormal with triple X chromosomes, its adenoviruses transformed and cultivated in the presence of 10% foetal calf serum, and whether this affects LHR function is unknown.

Other studies have used granulosa lutein cells collected in connection with OPU from women undergoing OS. However, these cells have experienced a heavy downregulation of their LHR gene expression following ovulation triggering, reaching a nadir of only a few percent at OPU compared with that at the time of ovulation induction (47, 48). Once the ovulatory cascade is over, LHR reappears in the lutein cells. Therefore, LHR expressed on granulosa lutein cells is newly synthesised and may not reflect the density and function of the LHR present on immature GC located in intact follicles during the follicular phase.

Regulation of LHR expression involves intricate mechanisms, including post-transcriptional degradation of *LHR* mRNA mediated by the *LHR* mRNA binding protein (LRBP) as studied by Menon and co-workers through several decades (49–52). LRBP interacts with specific sequences in the coding region of *LHR* mRNA, promoting its degradation (52), and LRBP has been shown active in early stages of folliculogenesis, just prior to ovulation and in connection with downregulation of *LHR* mRNA in response to a preovulatory LH surge. Most studies were performed in rats, but cultures of human granulosa lutein cells confirmed a similar role of LRBP on *LHR* mRNA expression (53). The LRBP has been identified as a mevalonate kinase involved in ovarian sterol metabolism and cholesterol synthesis (54), and it has been hypothesised that proteins involved in ovarian sterol metabolism may also be involved in regulation of *LHR* mRNA in human granulosa lutein cells (55).

Taken together, information of the function of LHR in human granulosa lutein cells is substantial but information of the functional effects of LHR expression taking place on GCs from intact follicles during the second half of the follicular phase in the natural cycle and/or in connection with OS is limited. It is furthermore likely that GCs from the natural cycle will differ from those that appear during OS with a reduced density of LHR expression.

## Interpretation of the results from the Rainbow trial

The reduction in the number of oocytes and follicles and resulting embryo development and achievement of pregnancies in connection with administration of rhCG during OS suggested altered and reduced GC function during the follicular phase and OS, whereas new profiles of steroids were also observed (**Figures 2**–**4**). Remarkably, hormone profiles spilt up into two groups that behaved differently. 17OH-P_4_, androstenedione testosterone, and E_2_ followed a similar and expected pattern with increasing concentrations during the cause of OS and with numerically increasing concentrations in parallel to escalating rhCG dosing on each specific day (i.e., day 1, day 6, day 8, and end of stimulation and OPU) (**Figures 2**–**4**). In contrast, concentrations of P_4_, inhibin-A, and inhibin-B (only measured on the day of OPU) were higher in the placebo group at end of stimulation (inhibin-A and inhibin-B) and at day of OPU (P_4_). Noticeably, a negative correlation was found between the dose of rhCG administered and measured concentrations (9, 10).

Follicular fluid concentrations followed the pattern in circulation with a dose-dependent, highly significant association to the dose of rhCG administered (10, 11). In addition, there were no signs of FF concentrations reaching a plateau even with the highest concentration of rhCG administered, suggesting that no ceiling had been reached. The group with the lowest dose of rhCG administered had significantly higher FF concentrations of all hormonal parameters compared with the control group—except for P_4_. For P_4_, the control group showed numerically the highest concentration except for the 12-µg rhCG group.

The two patterns of hormone secretion following rhCG administration in the Rainbow RCT (increase of some and decrease of others) represent different effects of rhCG on TCs and GCs: The paradoxically reduced P_4_, inhibin-A, and inhibin-B concentrations correlate with an unexpected attenuation of GC function in response to the rhCG administered in a dose-dependent manner. These contrasts increasing concentrations of the directly TC-derived products, including 17OH-P_4_, androstenedione, and testosterone, which followed an expected dose-dependent increase with rising rhCG exposure. Concentrations in FF parallelled this picture and showed altered hormonal profiles within individual follicles. Thus, results from individual follicles confirmed those observed in circulation and could therefore not explain the reduced number of follicles with a diameter of 12–17 mm.

Markedly, E_2_ concentrations as a GC product on the other hand follow a similar profile and increase as that of TC-derived hormones except on the day of end of stimulation and on OPU, where E_2_ levels in the highest rhCG group started to decline. However, expression of aromatase (i.e., CYP19A1) and E_2_ production is exclusively confined to GCs, however, with both FSH- and LH-like activity inducing aromatase (56). The reason for this development is most likely explained by the aromatizing capacity of the GC compartment, which greatly exceeds that of the substrate availability reflected in the reduction of androgens (i.e., androstenedione and testosterone) in FF being reduced almost a thousand times in the late preovulatory phase (19). Furthermore, the rhCG dosing groups resulted in increased androgen production thereby enhancing substrate availability. Therefore, the aromatizing capacity of preovulatory follicles also on stimulation days 6 and 8, being mainly induced by FSH, may not be significantly reduced by a potential negative impact of rhCG.

The results of the Rainbow RCT differ from those of a small RCT in which increasing doses of hCG (i.e., 0, 50, 100, and 150 IU urine-derived hCG daily) together with a constant FSH administration was given to women undergoing a long agonist protocol. Here, a similar increase in circulation and in FF amongst steroids including P_4_, 17OH-P_4_, androstenedione, testosterone, and E_2_ was found (57, 58). Noticeably, at end of stimulation, the relative increase in P_4_ in women administered 4 µg rhCG/day (≈125–150 IU uhCG/day, comparable with the highest dosing group (i.e., 150 IU uhCG/day) in (57, 58)) compared with the placebo group is a modest 1.17-fold increase. In contrast, data from Thuesen and co-workers’ studies showed a greater increase (1.61-fold increase) between placebo and the 150-IU uhCG (57, 58). Notably, the increase in the TC-derived hormone testosterone is similar between the two studies (1.72-fold increase in the Rainbow trial versus 1.76-fold in (57, 58).

Combined with the observed reduction exclusively by GC produced Inh-B and Inh-A in relation to increasing rhCG dose at the end of stimulation, this suggests that rhCG molecules cause a diminishing GC activity.

This suggests that the two types of hCG molecules used (urine versus recombinant) stimulate different intracellular signalling pathways. Importantly, GCs and TCs respond in a different manner to the rhCG suggesting differences in the LHR response.

However, the Rainbow data also suggest that the LHR expression and/or activity is affected by the rhCG administration because although a weak positive association between the concentration of P_4_ and rhCG dosing is observed in circulation at end of stimulation (9), this picture is reversed following final maturation of follicles as observed in both serum and FF with significant negative associations to the rhCG dose. Interestingly, final maturation of follicles is performed by a bolus trigger (6.500 IU) of a different hCG molecule (i.e., Ovitrelle), which demonstrates that the LHR expressed on GCs in connection with final maturation of follicles reacts opposite of what would be expected by the use of Ovitrelle. Thus, the observed effects in the Rainbow trial appear to result from a specific action of the rhCG on GCs plus an effect of LHR expression and/or activity.

However, irrespective of what mechanism affects GC function, it is of utmost importance to notice that the LHRs expressed on TCs react differently than those expressed on the GCs with two different hCG molecules.

The observed effects could be explained by LHR density on TCs and GCs being different, which may affect hormonal response and receptor clustering. A potential different receptor density in TCs and GCs may result from either FSH stimulation of LHR expression on GCs or from different mechanisms of receptor downregulation between the two cell types. As both LH and FSH are well known to act as biased agonists inducing different responses, the observed effects could be explained by a combination of differences in the LHR constitution and the ligands itself.

Another potential mechanism may involve an altered synthesis of LRBP in response to relatively high concentrations of rhCG, which thereby may alter the LHR expression differently between the two cell types.

Theca cells constitutively express LHR, whereas GCs in the selected follicle acquire LHR expression after follicular selection. It could be hypothesised that different densities and clustering of LHR expression take place in the two cell types. There is currently, to our knowledge, no experimental evidence to support this hypothesis except for the Rainbow RCT. This highlights the importance of the Rainbow RCT as hypothesis generating and reflects that functional characteristics of human LHR *in vivo* are difficult to study in immature human TCs and GCs.

Therefore, a sound conducted clinical trial in which GCs and TCs display immaturity as they appear *in vivo*, during the follicular phase, now suggests that some types of LH-like activity may attenuate GC function in preovulatory follicles during OS, whereas TC function appears unaffected.

Taken together, irrespective of whether the new rhCG molecule exerts effects different from current available hCGs, the results from the Rainbow RCT suggests a yet undescribed difference in LHR function exists between human TCs and GCs in connection with OS.

Furthermore, there is no information on how OS affects the LHR density as compared with the natural cycle and whether LHR density differs between TCs and GCs. Collectively, this demonstrates a gap in our understanding of the effects of LH-like activity during OS, which available *in vitro* data are unable to address.

## Limitations: LH release from the pituitary occurs in bursts

LH release from the pituitary occurs in bursts with an interval of around 60–90 min (3). If LH is administered exogenously providing constant levels, LHR will be downregulated, and cells become unresponsive (3). Exogenous administered hCG results in constant levels without bursts, but it appears that LHR downregulation occurs as expected but does become upregulated again after a relatively short period of time. However, whether the function of the LHR is similar between these two situations is not fully determined and whether the density of LHR is affected is also unknown.

Furthermore, it cannot be excluded that the specific rhCG molecule tested in the Rainbow RCT possessed a 3D structure different from other hCG molecules, which was the underlying cause of the effects observed.

Furthermore, the considerations presented here only focus on the hCG molecule. As to whether any of the proposed mechanisms also apply in connection with the human LH molecule is unknown.

The Rainbow RCT trial only included women in the long agonist protocol which led to very low endogenous LH levels (59). To what extent women following an antagonist protocol, with higher levels of endogenous LH-activity, would respond in a similar way is not clarified with this study.

## Conclusions and new areas to be considered for advancement of OS

The results of the Rainbow RCT support a new hypothesis for understanding the impact of hCG activity on human follicular development during OS with exogenous gonadotropins, which obviously will need confirmation from independent studies.

The trial suggested that rhCG specifically affects and attenuates certain GC functions during OS, leading to a reduced number of intermediate follicles and failure to augment P_4_, inhibin-A, and inhibin-B production. Conversely, TCs showed an expected response to rhCG in terms of sex steroid production. This differential response between TCs and GCs to rhCG proposes previously unrecognised differences in LHR expression and function.

The mechanisms underlying these differences are not clarified but may involve variations in LHR synthesis, density, clustering, and possibly glycosylation. Additionally, differences in LHR downregulation when exposed to rhCG may explain the distinct responses observed between GCs and TCs. However, the marked reduced concentrations of P_4_, inhibin-A, and inhibin-B as a result of being exposed to the ovulatory dose of Ovitrelle suggests that the rhCG have attenuated LHR responsiveness throughout the follicular phase.

In addition, it cannot be excluded that observed effects are based on artefacts created in the cell line producing the rhCG (i.e., HEC-293) as compared with the CHO cell line, which, however, does not distract from the fact that the LHR expressed on GCs and TCs apparently responds differently.

To advance our understanding of the impact of LH-like activity during OS, several areas warrant further investigation:

1. Characterising and studying LHR functionality on human immature TCs and GCs both *in vivo* and *in vitro*.
2. Evaluating the effect of high FSH concentrations during OS on GC and TC LHR expression compared with natural cycles, using GCs and TCs collected prior to the trigger for final follicular maturation.
3. Assessing whether different FSH preparations differentially affect LHR expression prior to the trigger for final follicular maturation.
4. Investigating LHR expression and function in women with polycystic ovarian syndrome (PCOS) compared with normal women, as LHR expression occurs at smaller follicular diameters in PCOS patients and may contribute to the aetiology of condition.

## Data Availability

The original contributions presented in the study are included in the article/supplementary material. Further inquiries can be directed to the corresponding author.
